# The Need for Multidisciplinarity in Modern Medicine: An Insight into Orthopaedic Infections

**DOI:** 10.3390/microorganisms10040756

**Published:** 2022-03-31

**Authors:** Andrea Sambri, Michele Fiore, Sara Tedeschi, Massimiliano De Paolis

**Affiliations:** 1 Orthopaedics and Traumatology Department, IRCCS Azienda Ospedaliero Universitaria di Bologna, Via Massarenti 9, 40138 Bologna, Italy; michele.fiore@ior.it (M.F.); massimiliano.depaolis@aosp.bo.it (M.D.P.); 2 Infectious Disease Department, IRCCS Azienda Ospedaliero Universitaria di Bologna, Via Massarenti 9, 40138 Bologna, Italy; sara.tedeschi5@unibo.it

As knowledge broadens, clinical practice becomes more elaborate, resulting in a variety of subspecialties and advanced health services. Sectorization of skills, even within the same medical specialty, has been the most common response to the two main goals of modern medicine: the achievement of the highest-quality standards in disease management and the need to optimize healthcare costs.

This model can be effective in the treatment of monodisciplinary diseases. Indeed, in these cases, patient management can be optimized through the progressive acquisition of clinical competence by a centre or individual healthcare provider. However, the segmentation of areas of expertise and the fragmentation of services may lead to logistical complications in the clinical management of complex diseases involving several organs or requiring a cross-disciplinary approach.

The establishment of healthcare models that cross over different medical specialties through the creation of a multidisciplinary team dedicated to the management of a specific disease or condition is a possible response to the need to bring together several ultra-specialized skills, allowing alignment with evidence-based best practices. Indeed, healthcare is a multidisciplinary activity in which a variety of healthcare professionals from various specialties need to work together, communicate, and often share resources. A successful multidisciplinary approach is therefore a fitting model of a healthy health service that optimises resources to produce the most comprehensive assessment of a patient’s condition and provide a well-rounded treatment plan.

The possibility of an integrated healthcare has progressively gained attention in recent decades, although the principle of the multidisciplinary approach was already introduced at the beginning of the 20th century by the Mayo brothers: “It has become necessary to develop medicine as a cooperative science; the clinician, the specialist, and the laboratory workers uniting for the good of the patient, each assisting in elucidation of the problem at hand, and each dependent upon the other for support.”—William J. Mayo, Commencement speech at Rush Medical College, 1910.

Gathering health professionals from different backgrounds working for the same objective can positively influence patient outcomes but can also relieve the pressure on health professionals themselves. There are several potential advantages associated with a multidisciplinary approach that can be experienced at multiple levels. The hospital is more likely to increase the volume of patients and patient referrals, thereby increasing the experience gained by clinicians in treating the specific condition. Individual doctors will be in contact with a larger number of cases, also improving their knowledge and technical skills through participation in multidisciplinary discussions, giving the patient a greater chance of receiving coordinated and personalized care. A multidisciplinary team may also have the potential to develop and promote community health initiatives to encourage education on disease-prevention behaviours among patients.

In orthopaedics, the most striking example of multidisciplinarity is probably provided by the management of the polytraumas. Appropriate diagnostic and therapeutic approaches to the polytrauma patient require efficient trauma management systems and integrated teams [1]. The best approach involves anaesthesiologists, trauma surgeons, diagnostic and interventional radiologists, general surgeons, urologists, and neurosurgeons, among others. Good dialogue between team members on important clinical pitfalls, continuous vigilance by all healthcare providers involved, and centralised care planning are essential [2].

However, the need for multidisciplinary management in orthopaedics does not end with polytrauma. In fact, one of the earliest adopters of a multidisciplinary teamwork was the field of orthogeriatrics (trauma in elderly patients), where patient management is shared between internists and surgeons [3]. Due to the ageing of the surgical population, surgical safety for frail patients has become a critical issue. Evidence shows that geriatric co-management of elderly orthopaedic patients results in improved function, shorter length of stay, and fewer complications [4]. Historically, the multidisciplinary approach has also significantly improved the care of oncologic patients. It has been shown that both the time to diagnosis and clinical outcomes have a positive influence when multidisciplinary case management is effective [5]. An impressive network of specialists is also required for the oncological field in orthopaedics, including orthopaedic surgeons, medical oncologists, pathologists, radiation oncologists, and frequently general surgeons, vascular surgeons, plastic surgeons, urologists, and gynaecologists. The latter specialized surgeons are often required, especially in the management of patients undergoing pelvic surgery, not only in the field of oncology, but also in the case of prosthetic revision surgery, or in the aforementioned management of polytrauma patients [6].

In addition to these areas, the need for a multidisciplinary approach is also emerging in the field of musculoskeletal infections. The management of these infections is very challenging, as diagnosis often is based a combination of clinical signs/symptoms and laboratory and imaging findings, and treatment usually requires prolonged antimicrobial treatment and major surgical procedures. Moreover, the number of patients with bone and joint infections is expected to increase, due to the ageing population and the progress in surgical techniques in joint replacement and fracture fixations, among others.

To date, there is no strong evidence in the literature of benefits of a multidisciplinary management of musculoskeletal infections, yet the principles of diagnosis and factors influencing of patients with infections are similar to those of previously mentioned settings (such as polytrauma, orthogeriatrics, orthopaedic oncology, etc.). Thus, it seems intuitive that a similar approach to treatment could produce similar improved outcomes.

Indeed, as is widely known, the treatment of musculoskeletal and joint prosthesis infections represents a real challenge for patients, healthcare providers and the healthcare system itself, because of the high number of treatment failures and the high economic burden of managing these diseases [5]. Continuous collaboration between skilled orthopaedic surgeons, infectious diseases specialists, and microbiologists, at a minimum, should be the cornerstone for optimizing the management processes of all musculoskeletal infections. However, for some very complex cases, a further multidisciplinary integration is desirable, or even essential, in both clinical and pre-clinical settings. Therefore, plastic surgeons, vascular surgeons, and general surgeons may be asked to provide a primary contribution to the clinical assessment and treatment choices, which is often personalized. At the same time, clinical pharmacologists and pathologists can play a key role in clinical decision making and contribute to the introduction of new methods and techniques.

In particular, in the multidisciplinary context of the management of musculoskeletal infections, it is possible to recognize the specific contributions primarily (but not exclusively) of:

- Orthopaedic surgeons: they have a central role in the diagnosis and represent the key figure of the multidisciplinary team, as a surgical treatment is required in the management of most patients with musculoskeletal infections. They are required to have specific expertise in the management of infections in order to be able to perform an appropriate surgical treatment.
- Infectious disease specialists: they share with orthopaedic surgeons a critical role in the diagnostic process and lead the management of antibiotic therapy in terms of drug selection, treatment duration, and monitoring for safety and efficacy.
- Microbiologists: their contribution is crucial to establish etiological diagnosis of infections. The role of musculoskeletal microbiology is rapidly evolving with developments in nucleic acid sequencing-based techniques for diagnosis, for which the microbiologist enables the interpretation of results [7].
- Radiologists and Nuclear medicine specialists: they may contribute to the diagnostic phase on the basis of morphological and functional features of the infectious processes [8]. Moreover, imaging techniques allow information to be acquired about the extension of the infectious process and the involvement of adjacent structures that can be very important to the surgical phase.
- Clinical pharmacologists: through their expertise with the pharmacokinetics and diffusion of antibiotics into the different tissues, they can contribute to the drug-selection process and optimization of administration. In addition, further input can be offered by the pharmacologist regarding the release of antibiotics from bone substitutes and cements, the use of which is very common in the treatment of osteomyelitis and prosthetic joint infections.
- Plastic surgeons: they may provide a crucial contribution in cases where the extension of the infectious process would not allow sufficient coverage of deep tissues. Plastic reconstruction techniques in many cases allow valid alternatives to amputation of the limb.
- General surgeons, vascular surgeons, urologists, gynaecologists and neurosurgeons: these specialists may be involved in selected cases of very complex interventions requiring accessory additional surgical procedures.
- Non-physician specialists such as physiotherapists and specialized nurses should not be ignored, as rehabilitation and wound care are an essential part of the management of the patient with an osteoarticular infection.

All specialists should be involved in all stages of the management pathways, including diagnosis, treatment (both surgical and non-surgical), and long-term follow-up.

In the field of musculoskeletal infections, the most representative example of a specific area that could benefit from a systematic and structured multidisciplinary approach is probably the management of periprosthetic infections (PJI). PJI affects about 0.5–3% of patients undergoing total joint arthroplasty [9,10]. The economic burden to the healthcare system of treating a single case of PJI can be very high. Patient burdens are even higher, with a long hospital stay, numerous surgeries, related pain and suffering, disability and impaired quality of life, and risks of surgical morbidity and mortality. Since the moment of diagnosis, the management of PJI remains controversial and complex. PJI may present different forms and stages from the time of arthroplasty. After the diagnosis is established, patients usually need a series of major surgical procedures (for which orthopaedic expertise may not be sufficient), combined with antimicrobial treatment over several weeks, which is highly individualized based on the type of microbial population and patient characteristics, as well as any comorbidities.

It may be difficult for an individual surgeon to appropriately assess a patient with a painful arthroplasty and to appropriately choose and monitor an antibiotic therapy; on the other hand, it is impossible for infectious disease specialists to effectively cure a patient with a PJIs only with antibiotics.

One option for dealing with this problem is to manage these challenging patients with a multidisciplinary team. Indeed, the recurrence of PJIs is high and is reported to be between 8% and 70% [11], and complications associated with surgery are common. Thus, it is evident that similar factors have been recognized in other aspects of orthopaedic surgery, and it is recognized that optimized results are derived from a multidisciplinary approach to management [12,13].

Management challenges similar to those described for PJIs are also common among other musculoskeletal infections. Acute and chronic destructive osteomyelitis, septic arthritis, and soft tissue infections, including non-bacterial infections, infections after fracture fixation, spondylodiscitis, etc., can all strongly benefit from multidisciplinary collaboration, as many different procedures may be required, and patient needs may vary from case to case.

Whatever is valid from a clinical point of view is equally valid from a scientific point of view. Progress in the field must necessarily involve boundary issues between different specialties and therefore requires strong multidisciplinary collaboration. There is a strong need to investigate clinical or pre-clinical and methodological aspects that require multidisciplinary management, because it is in these areas of research that progress is most likely to lie. We strongly believe that only the increased dissemination of knowledge can contribute to the optimization of the management of such complex matters. We therefore hope that more and more research will be conducted on these topics in the future.
